# Finite Element Analysis of Osteosynthesis Screw Fixation in the Bone Stock: An Appropriate Method for Automatic Screw Modelling

**DOI:** 10.1371/journal.pone.0033776

**Published:** 2012-03-28

**Authors:** Jan Wieding, Robert Souffrant, Andreas Fritsche, Wolfram Mittelmeier, Rainer Bader

**Affiliations:** Department of Orthopaedics, University of Rostock, Rostock, Germany; University of California at Berkeley, United States of America

## Abstract

The use of finite element analysis (FEA) has grown to a more and more important method in the field of biomedical engineering and biomechanics. Although increased computational performance allows new ways to generate more complex biomechanical models, in the area of orthopaedic surgery, solid modelling of screws and drill holes represent a limitation of their use for individual cases and an increase of computational costs. To cope with these requirements, different methods for numerical screw modelling have therefore been investigated to improve its application diversity. Exemplarily, fixation was performed for stabilization of a large segmental femoral bone defect by an osteosynthesis plate. Three different numerical modelling techniques for implant fixation were used in this study, i.e. without screw modelling, screws as solid elements as well as screws as structural elements. The latter one offers the possibility to implement automatically generated screws with variable geometry on arbitrary FE models. Structural screws were parametrically generated by a Python script for the automatic generation in the FE-software Abaqus/CAE on both a tetrahedral and a hexahedral meshed femur. Accuracy of the FE models was confirmed by experimental testing using a composite femur with a segmental defect and an identical osteosynthesis plate for primary stabilisation with titanium screws. Both deflection of the femoral head and the gap alteration were measured with an optical measuring system with an accuracy of approximately 3 µm. For both screw modelling techniques a sufficient correlation of approximately 95% between numerical and experimental analysis was found. Furthermore, using structural elements for screw modelling the computational time could be reduced by 85% using hexahedral elements instead of tetrahedral elements for femur meshing. The automatically generated screw modelling offers a realistic simulation of the osteosynthesis fixation with screws in the adjacent bone stock and can be used for further investigations.

## Introduction

Large segmental defects and non-unions in long bones caused by fracture, infection, tumour or cysts are still a challenging problem in orthopaedic surgery. The stable fixation of an osteosynthesis system is necessary for the bone healing process and the clinical success of the implant. Manufacturers worldwide developed various methods to offer maximum intraoperative flexibility (e.g. polyaxial screws) and stable screw-plate connection (e.g. angular stable fixations) [1], [2]. The functionality of the mentioned fixation methods has been demonstrated in several experimental studies [3]–[5]. Nevertheless, experimental testing is often time-consuming, cost-intensive and accurate results have to be extracted with extensive equipment.

Besides experimental testing, finite element analysis (FEA) has grown to a powerful tool in order to analyze stresses and strains within structures during static and dynamic load situations. Moreover, it offers detailed information which cannot be determined with experimental methods. Due to the capability to analyse the influence of various parameters on implant components during the preclinical testing, without prototype production, the FEA has become an irreplaceable tool with various applicability. Therefore, it is a common method in mechanical engineering and gains more and more influence in biomechanics.

Furthermore, interactions of the plate-screw-bone composite could be implemented and analyzed in computational simulations [6]–[8], whereat fixation of osteosynthesis implants like plates or nails to the bone stock can basically be considered with three different numerical approaches.

First, there is the simple approach to fix an implant directly to the bone by a tied contact [9]. In this case the outer surface nodes of the bone are tied to the reference surface. Thereby, translational degrees of freedom (DOF) of the bone are associated with the corresponding DOF of the implant and no relative displacement between the nodes and subsequently no elastic deformation can occur. This may results in artificial stiffening of the contact area and deviant stress and strain distributions within the contact area. In addition, stresses within the bone can differ, because no elements for the screws were considered. Furthermore, contact forces are only transmitted via the outer surface.

Second, there is a common approach to model the screws with three-dimensional solid elements. This is a frequently described method which considers the existence of screws by adding cylindrical shape to a model to approximate the geometry of the screws. This method is used for different applications, e.g., intramedullary nails [6], [10] or plates for oral and maxillofacial surgery [11], [12], spine surgery [13], [14] and especially for bones [6]–[8], [15], [16]. To realize the fixation, the cylinders are mostly meant to be in perfect geometric contact to the bone stock and fixed to it. This method requires the modelling of the cylindrical screws as well as consideration of the drill holes within the bone model prior to meshing. Therefore, fine meshing around the drill holes has to be performed in order to preserve a round curvature, generating enough elements for a realistic mechanical behaviour and subsequently an adequate stress transfer between bone and screw. Relative displacement between the bone and osteosynthesis system is still enabled and loading forces are induced via the screws into the bone.

Third, there is the advanced approach, in our point of view, which implies the usage of structural elements, i.e. beams, for screw modelling [10], [17], [18]. The fact, that this method could be used without considering the screw holes and mesh densities of the contact area during the meshing process is the major benefit of this technique. Furthermore, these two-dimensional elements provide an excellent mechanical behaviour and could be used to model the screws and the connections to the three-dimensional elements of the bone. Even the analysis of an equivalent osteosynthesis system is possible with this technique [19]. This approach decreases the computational costs for the analysis but at the same time increases the modelling effort for the screws and their connections to the bone.

The aim of the present study was to demonstrate the benefit of an automatic screw modelling with structural elements and its fixations with an adequate accuracy on arbitrary FE meshes. In addition, this method was compared to experimental testing and other frequently used modelling approaches. Furthermore, the importance of an appropriate fixation technique, exemplarily in case of large contact areas between bone and implant, has been investigated.

## Materials and Methods

### Generating finite element models

CAD models of a composite femur (4th generation large left, Sawbone Europe AB, Malmö, Sweden) and an angular-stable osteosynthesis plate (NCB®, Zimmer GmbH, Freiburg, Germany) were generated from CT data with an approximate voxel size of 0.6 mm cube. The general procedure was described in a previous work of Kluess, et al. [20]. A segmental bone defect of 30 mm in the distal diaphysis of the femur was created analogue to the later illustrated experimental setup prior to the meshing of the femur. Convergence testing with respect to the femoral deflection was performed in order to avoid any influence of the mesh density on the results. Meshing of the bone was generally performed neglecting the differentiation of bony structures and the elements exhibited a global edge length of approximately 2 mm. Edge length and degree of freedom (DOF) for the femur implies being in good convergence [21], [22].

**Figure 1:**
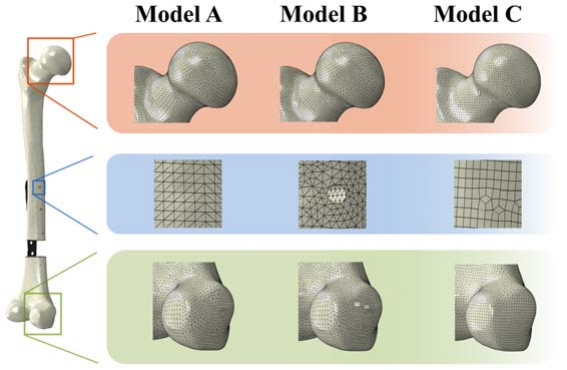
Magnification of the mesh for the three different femoral models. Magnifications are given for the area around the femoral head (red box), the area around the screw (blue box) and the area of the medial condylus (green box). Thereby, Model A and B consisted of tetrahedral elements, whereat Model A did not consider any screw holes. Model C was discretised with hexahedral elements and did not consider any screw holes, either.

Due to the different screw modelling and fixation techniques, meshing procedure of the femur was performed in three work steps using mainly the FE software package Abaqus (Version 6.10 EF, Dassault Systèmes, Vélizy-Villacoublay, France). Thus, three different numerical models for the femoral bone were generated as illustrated in Figure 1.

The first femur model (Model A) was meshed with ten-node second-order tetrahedral elements (T10) without taking into account any screw holes. This model was used for the tie contact as well as for the structural screw model and consisted of 227,000 elements.

The second femur model (Model B) was also meshed with T10 elements but considered the screw holes with a diameter of 4 mm for the later implemented solid cylindrical screws. The holes were cut into the bone, correlating with the direction vectors of the screws according to the experimental test setup. This model consisted of 274,000 elements.

A further femur model (Model C) was meshed with eight-node first-order hexahedral elements (H8) analogue to the first tetrahedral model (Model A) using the FE software package HyperMesh (Version 10.0, Hyperworks, Altair Engineering GmbH, Böblingen, Germany). Screw holes were not considered because they were also modelled as structural elements. This model was used to compare the influence of different element types on the result accuracy and the computational time and consisted of 111,000 elements.

The osteosynthesis plate was meshed with eight-node hexahedral elements (H8; 14,000 elements), using the FE software package MSC.Patran (Version 2007r2, MSC Software Corporation, Santa Ana, CA, USA).

Besides the illustration of the different femur meshes, number of elements and element type for each model are listed in Table 1.

**Table 1:** Summary of the investigated models.

Femur-Model	Part	Element type	Number of elements
Model A	Femur	C3D10	227,000
	NCB	C3D8	14,000
Model B	Femur	C3D10	274,000
	NCB	C3D8	14,000
Model C	Femur	C3D8	110,000
	NCB	C3D8	14,000

For each investigated femoral model element types and number of elements are listed. The osteosynthesis plate (NCB®) was the same for each model.

### Material properties

**Figure 2:**
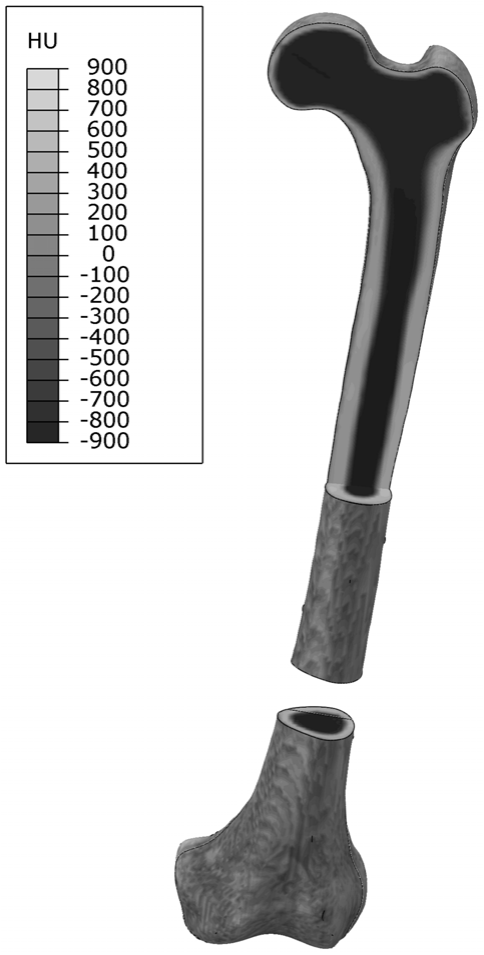
Distribution of the material properties along the femur. Femur with its segmental defect at the lower third is shown. An additional view cut in the frontal plane was created at the proximal end to show the cortical structure along the femoral axis. Dark colour represents areas with low HU values, e.g. air and cancellous bone, light colour represents areas with high HU values, i.e. cortical bone.

Bone was modelled as linear elastic and isotropic material with an inhomogeneous material distribution. Inhomogeneity of the bone was derived from CT data and was applied as a function of the Hounsfield (HU) values [20] as illustrated in Figure 2. Elastic constants were taken from the manufacturer's data with a Young's modulus of 16.7 GPa and 137 MPa for cortical and cancellous bone respectively. The Poisson's ratio was assumed to be 0.3.

All other materials were modelled as linear-elastic, isotropic and homogeneous. Material properties of the titanium alloy (Ti6Al4V) for both plate and screws were derived from the manufacture's data and from literature [6], [20], [23] with a Young's modulus of 110 GPa and a Poisson's ratio of 0.3.

### Fixation of the osteosynthesis plate to the bone stock

FE meshes of plate and femur were assembled within the FE software package Abaqus for further usage and screw modelling. Femur and plate were positioned according to the experimental testing. Based on the three femur models, five different cases for fixation were developed and listed in Table 2.

**Table 2:** Overview of the investigated fixation cases.

	Case				
	A1	A2	B	C	D
Femur model	A	A	B	A	C
Element type of the femur	C3D10	C3D10	C3D10	C3D10	C3D8
Number of elements	227,000	227,000	274,000	227,000	110,000
Fixation method	Tied (2)	Tied (7)	Solid	Structural	Structural
Screw	—	—	Cylindrical volume	Beam	Beam
Number of screw elements	—	—	8,500	2,300	1,900
Element type of screw	—	—	C3D10	B32	B32
Screw holes	No	No	Yes	No	No

Five different cases for the three fixation methods are investigated, based on the three different femur models. Furthermore, information of the implemented screws is also provided.

In case of the first fixation method no screw modelling or screw holes were necessary (Case A1). The nodes of the medial (inner) side of the NCB® plate were tied to the outer nodes on the lateral side of the femur (Model A) in two separate contact areas, proximal and distal of the segmental gap. Subsequently, no relative displacement between the femur and the plate could occur. To reduce an unintended stiffening of the contact zone, a subset of this fixation method was investigated (Case A2). Thereby, the contact area was separated into seven areas, beneath and adjacent to the screws holes of the plate.

For the second fixation technique (Case B) seven solid screws, dicretised by solid elements (T10), were implemented into screw holes of the femur (Model B). The geometry of the screws exhibited a cylindrical shape with an outer diameter of 4 mm and a length of 40 and 80 mm, respectively for five cortical and two cancellous screws. Fixation between femur and screws was modelled by direct mesh connectivity. In such a case elements of the neighbouring materials share the same nodes and could not be separated from each other. Translational DOF of all screw head nodes were bonded to the nodes of the corresponding holes of the plate representing an angular-stable fixation.

**Figure 3:**
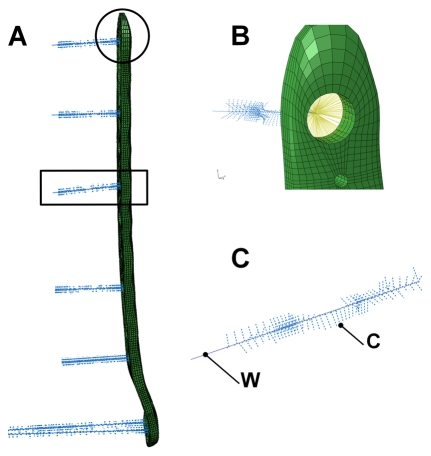
Osteosynthesis plate and structural screws. FE model of the osteosynthesis plate and the seven generated screws (a) (view from anterior, see (b) and (c) for detailed illustration). Angular stable fixation of the screw heads to the nodes of the osteosynthesis plate (b) (view from lateral). Screw with the rigid connector elements C between the base points on the wire W and the FE elements of the bone (c).

The last fixation method used structural elements to model the screw and the connection to the three-dimensional FE elements of the bone (Case C: Model A, T10 and case D: Model C, H8 mesh) Furthermore, Abaqus/CAE offers the possibility to automatise processes by a scripting interface, which uses the higher-level program language Python. Python version 2.5, comprised by the used Abaqus edition, was enhanced by the mathematical NumPy package (Version 1.2.1). Structural screws were modelled by an interactive script according to five requested parameters, i.e. screw head coordinates, direction vector, length, root diameter and outer diameter. Information about midpoints of the screw heads and direction vectors for each screw were determined from CT data of the experimental test setup. The endpoint of each screw was calculated by length and direction vector and connected to the midpoint of the corresponding screw head by modelling a wire penetrating the elements of the femoral bone (Figure 3). FE nodes of the femur in the proximity of the screw wire and at distance less or equal to the outer screw radius, i.e. 2.5 mm, were connected perpendicular to the wire using rigid connector elements (Figure 3 c). The calculated intersection points between wire and connector elements were subsequently used for meshing the wire with beam elements (B32). The circular cross section of the beam elements were designated with a radius of 2.0 mm, the effective root diameter of the thread. Angular stable fixation of the screw head was implemented by coupling the rotational and translational DOF of the node representing the position of the screw head with the DOF of the nodes within the screw hole of the plate (Figure 3 b).

### Loads and boundary conditions

The femur was loaded with an axial load of 227 N according to the experimental setup, while the translational displacements at the distal nodes of the femur were inhibited. Load was transferred into the femur by a frictionless contact between a rigid plate and the femoral head.

### Experimental test setup

Experimental testing was conducted using a composite femur (4th generation large left, Sawbone Europe AB, Malmö, Sweden) and an angular-stable non-contact bridge (NCB®, Zimmer GmbH, Freiburg, Germany) of medium size (246 mm length, 16 screw holes) and made of standard titanium alloy (Ti6Al4V). This osteosynthesis system consists of threadless screw heads for a polyaxial screw positioning within the plate. Angular-stable fixation is guaranteed by application of an additional locking cap. Size and position of the plate and screws were chosen by an experienced surgeon to fit the clinical situation.

**Figure 4:**
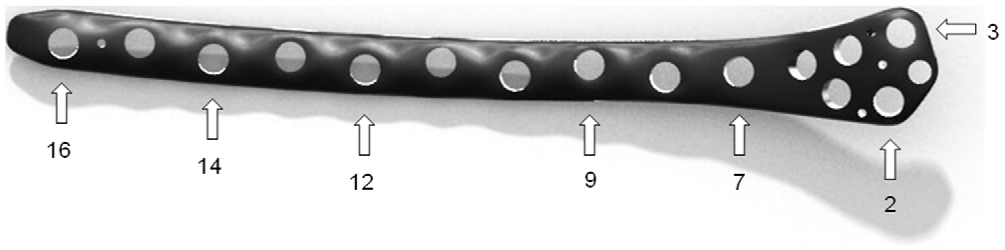
CAD model of the plate as a schematic figure for the screw position. Arrows marking the position at which screws are placed for the fixation to the bone.

The NCB® plate was fixed to the bone prior to the segmental defect creation to ensure the correct position of the femoral bone fragment with respect to the intact situation. Seven holes (4 mm diameter) at the positions 2, 3, 7, 9, 12, 14 and 16 of the plate (marked with arrows in Figure 4) were drilled into the femur. The positions were chosen to provide an adequate fixation of the bone. Two cancellous screws (80 mm length) at position 2 and 3 and five cortical screws (40 mm length) at the remaining five positions were used, following angular-stable fixation with locking caps. All screws were made of the same titanium alloy as the plate, exhibited an outer diameter of 5 mm and were tightened with a defined torque of 6 Nm.

**Figure 5:**
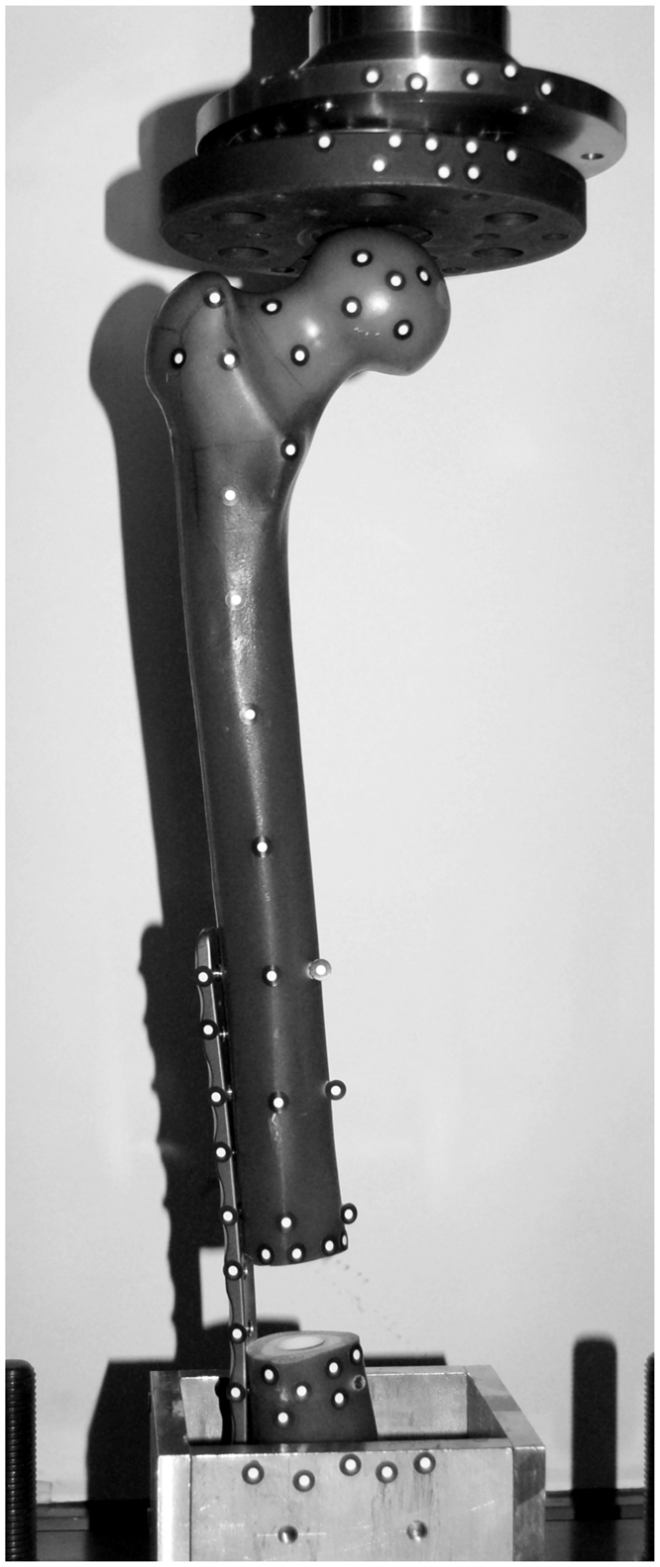
Experimental test setup. Posterior view of the test arrangement with a composite left femur mounted in the universal testing machine (Zwick/Roell). Segmental defect is bridged with an osteosynthesis system on the lateral (outer) side and fixed with seven titanium screws. Distal end of the femur is embedded in a metallic socket, filled with casting resin. 57 optical markers were attached onto the femur, socket and the testing machine to calculate their displacement during loading.

Finally, a 30 mm segmental defect was created 120 mm proximal to the condyles of the femur and parallel to the knee joint axis. 70 mm of the distal end of the femur and subsequently parts of the plate were fixed with polyurethane casting resin into a metallic socket for mounting into a universal testing machine (Zwick Roell, Z050, Ulm, Germany) (Figure 5). To guarantee an axial load on the femoral head, a bearing inhibiting the transmission of shear forces was placed between the femoral head and the crosshead of the testing machine. Furthermore, 57 optical markers were attached to the surface of the femur, the proximal and distal defect edges and the osteosynthesis plate in order to determine the femoral displacement and the gap alteration. Three-dimensional displacements of the markers were calculated using the dynamic stereo-image based motion analysis system Pontos (5 M, GOM mbH, Braunschweig, Germany).

A maximum load of 227 N (17 N for the bearing, 10 N for preloading and additional 200 N by the testing machine) was applied onto the femoral head to avoid plastic deformation of the NCB® plate. Three loading procedures have been performed in order to average any variability. Mean values and standard deviations (SD) were calculated.

## Results

Accuracy of the optical measuring system was determined with two markers at the rigid metallic socket and showed an error of 2.9±3.8 µm (mean value and SD). Hence, it did not have a measurable influence on the results.

**Figure 6:**
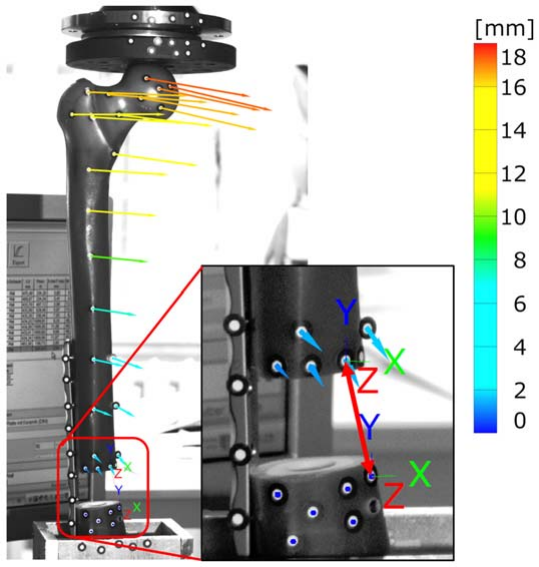
Results of the optical measurements. Displacement vectors calculated for each marker along the femur at a maximum applied load of 227 N. Direction and magnitude are plotted in the picture taken from the optical measuring system at the last loading step. Calculation of the gap alteration was calculated with displacement vectors between two marker points (indicated with red double-sided arrow). The colour legend represents the displacement magnitude.

Displacement vectors for each optical marker during loading were calculated representing the deflection of the bone. Magnitude and direction for all markers of the femur are shown in Figure 6 for the last frame of the testing procedure, i.e. a load of 227 N. The displacement of the femoral head as well as the alteration of the gap distance under the applied load was the most important factor for comparison. Mean value and standard deviation of the load-displacement relation were calculated of all testing procedures. A mean deflection of 16.99±0.16 mm occurred for the midpoint of the femoral head marker. Gap alteration was determined between markers at the proximal and the distal end of the defect with a magnitude of 1.78±0.02 mm. Only small deviations of the results between the measuring procedures occurred.

**Figure 7:**
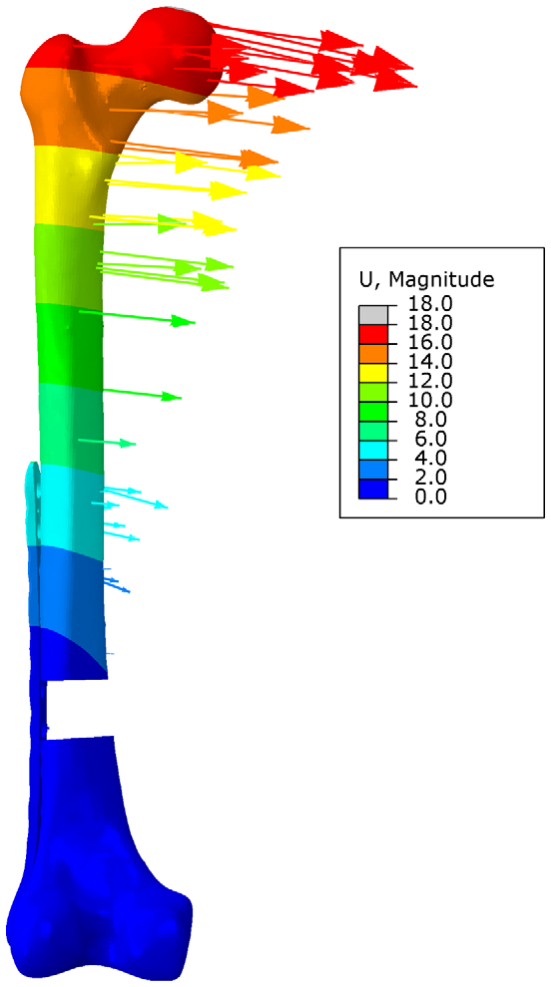
Displacement magnitude |U| of the numerical analysis. Displacement field of the femoral bone and displacement vectors for individual nodes (not equal to those of the experimental test setup) are shown.

The resulting displacement magnitude |U| for the full femoral bone, as calculated by numerical analysis, is shown in Figure 7. Furthermore, displacement vectors for single nodes (without correlation to the optical markers) are plotted, indicating the direction and the magnitude of the displacement. Both magnitude and direction of the numerical analysis were close to the results obtained from experimental testing.

**Figure 8:**
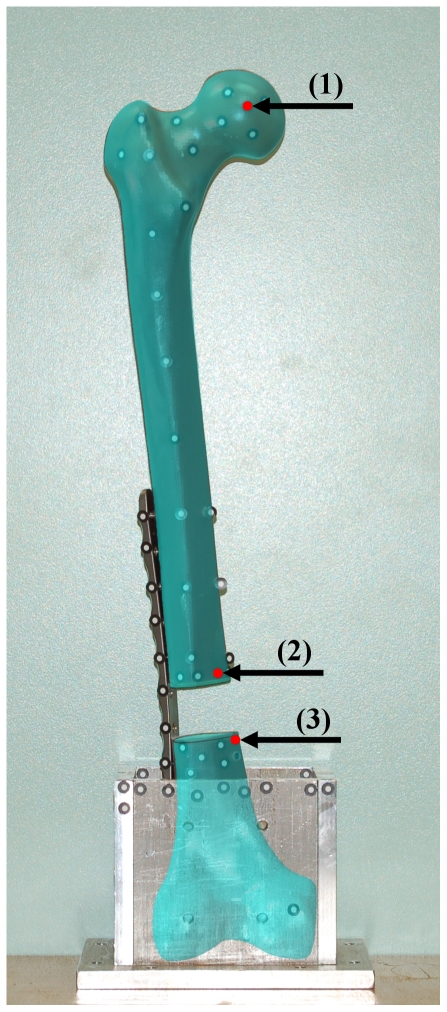
Alignment of the marker points to the FE model. Overlay plot of the test setup picture with the marker points and the FE model within the FE software package. By using the translucency for the FE model the position of the marker points could be adapted to the FE model. Red marks show position of the nodes, used for the calculation of the femoral head deflection (1) and for the gap alteration (2 and 3).

For exact comparison of the numerical results with the experimental testing, the position of three markers (one for deflection of the femoral head, two for gap alteration) were transferred to the numerical models and subsequently used for the displacement calculations (Figure 8). The positions of the three points were the same for all numerical models.

**Figure 9:**
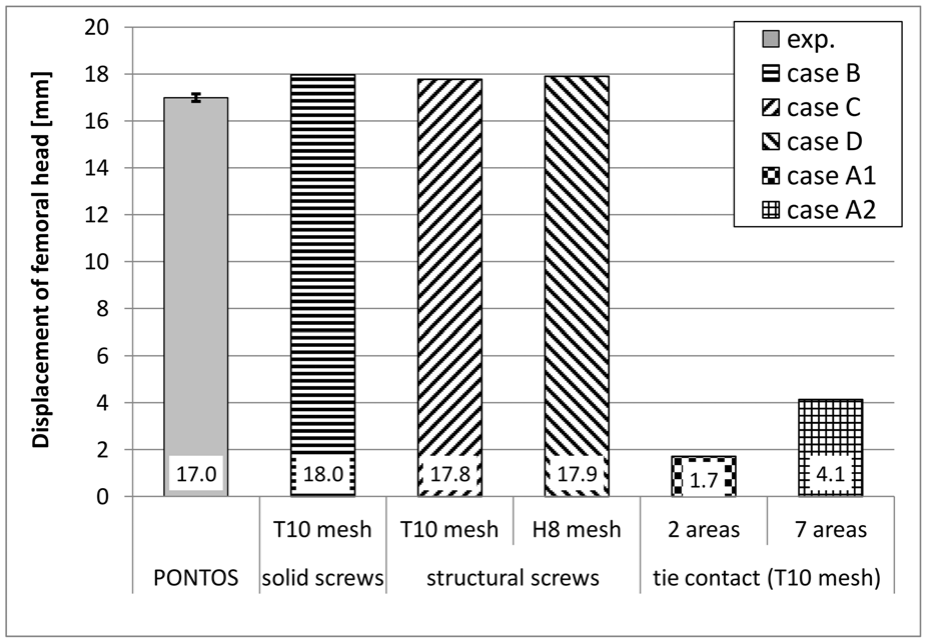
Results for the femoral head displacement. Results were obtained from the experimental testing (Pontos) and the five numerical analyses. Mean value and standard deviations are shown for the experimental data, while magnitudes of femoral head displacement are shown for the data of all numerical models. All results were achieved at a load of 227 N.

Femoral head displacement of the experimental testing and the numerical analysis are plotted in Figure 9. Good agreement could be shown between the experimental results and the numerical models concerning the screws (Case B to D). For these FE models the results were approximately 5% larger than those obtained from experimental testing. Deviation between the model with solid screws and both models with the structural screws were 1.0 and 0.3% for the tetrahedral and the hexahedral models respectively. Differences between the two structural screw models were less than 1%. In contrast, results for both models with the tied contact methods (Case A1 and A2) showed deviations of more than 75% of the experimental results. Although the tied contact was performed with seven individual contact pairs and created in the adjacent area of the screws only, the deflection of the femoral head, calculated with 4.1 mm, was too small and showed only 25% of the deflection measured experimentally. The result for the case A1, with a decrease of 1.7 mm, was even worse and only 10% of the displacement obtained experimentally. Furthermore, differences between both tied fixation models were more than 140% between the cases A1 and A2.

**Figure 10:**
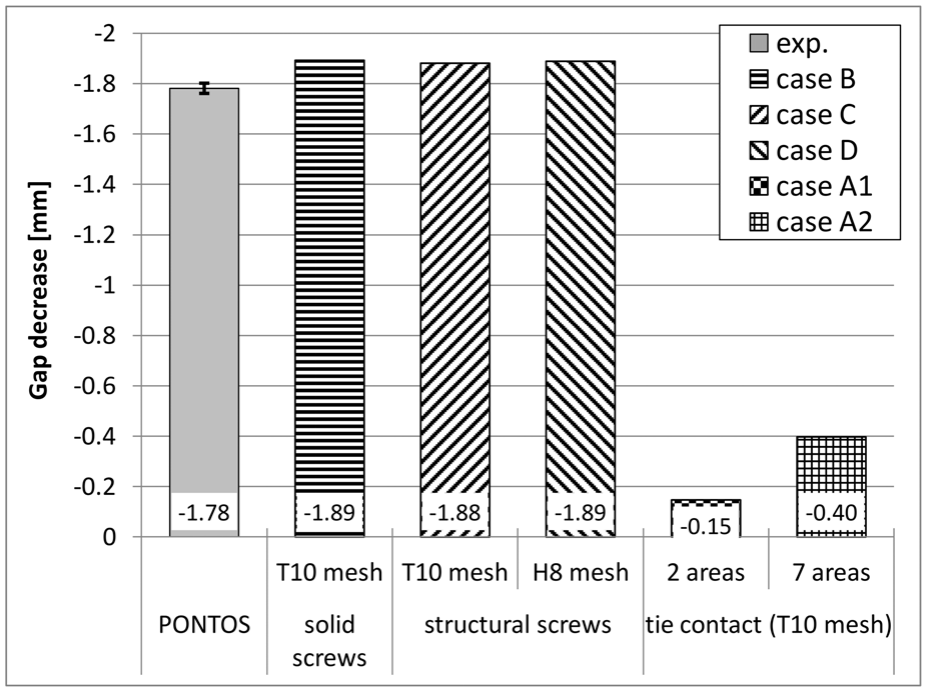
Results for the gap decrease. Results were obtained from the experimental testing (Pontos) and different numerical analyses. Mean value and standard deviations are shown for the experimental data, while magnitudes of gap width are shown for the numerical data of all five numerical models. All results were achieved at a load of 227 N.

Figure 10 shows the comparison between the experimental tests and the numerical analysis for the maximum gap alteration. Again, good agreement could be determined between the experimental results and the numerical screw models. Results for the screw models were approximately 6% larger than the experimental results, whereas the deviation between the structural screw and the solid screw models was less than 0.6%. Variability between both structural screw models were less than 0.5%. Results of the two and the seven tie fixation models were respectively 92 and 78% less than the experimental results. Deviation was more than 170% for the case A2 compared to the case A1.

**Figure 11:**
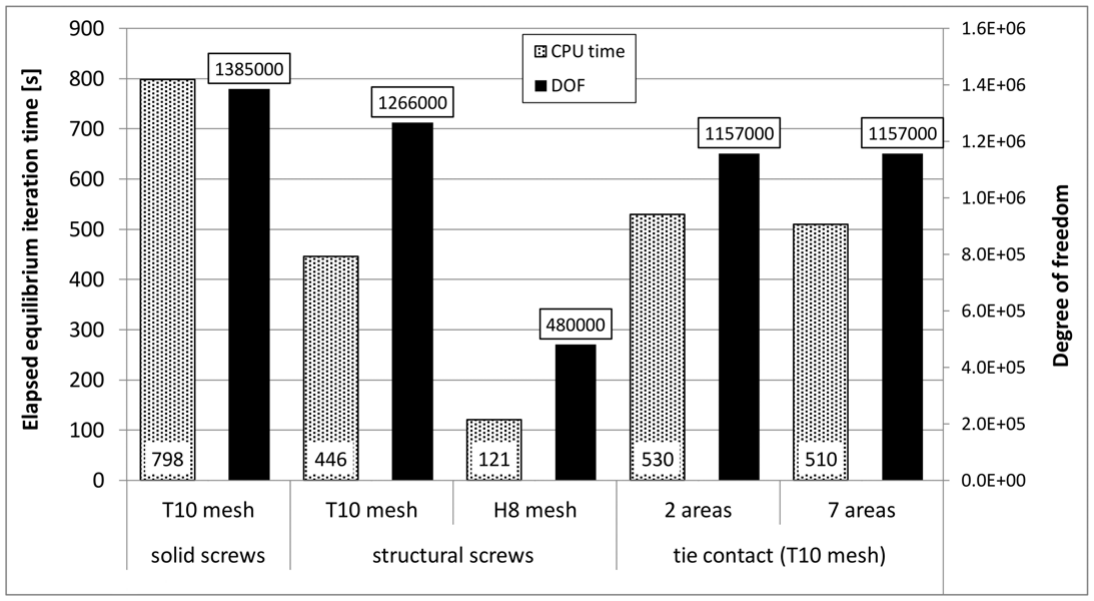
Elapsed total CPU time for one equilibrium iteration. For all five numerical models the calculations were performed on an Intel® Xeon® processor E 5504 2.0 GHz. Elapsed time for one equilibrium iteration and the DOF for each model are shown. Least DOF and subsequently least computational time was determined for the hexahedral model.

Besides the comparison of the result accuracy, the computational time for all numerical models was analyzed and plotted (Figure 11). All calculations have been performed on an Intel® Xeon® processor E 5504 2.0 GHz. In order to take the different fixation types into account, only the elapsed CPU time for one equilibrium iteration was used for comparison. For the T10 numerical model (Model B), concerning the solid screw elements, the highest computational time was necessary. This model also exhibited with a value of 1,386,000 the largest amount of DOF. Although the amount of DOF for the T10 model with the structural screws (Model A) was only 8% larger (1,266,000 DOF) than for both tie fixation models (also Model A: 1,157,000 DOF), due to the structural screws, the computational time was approximately 17% less. Least computational time was necessary for the H8 model (Model C) with only 480,000 DOF. The computational time of model C was only 15% of the computational time compared to Model B.

**Figure 12:**
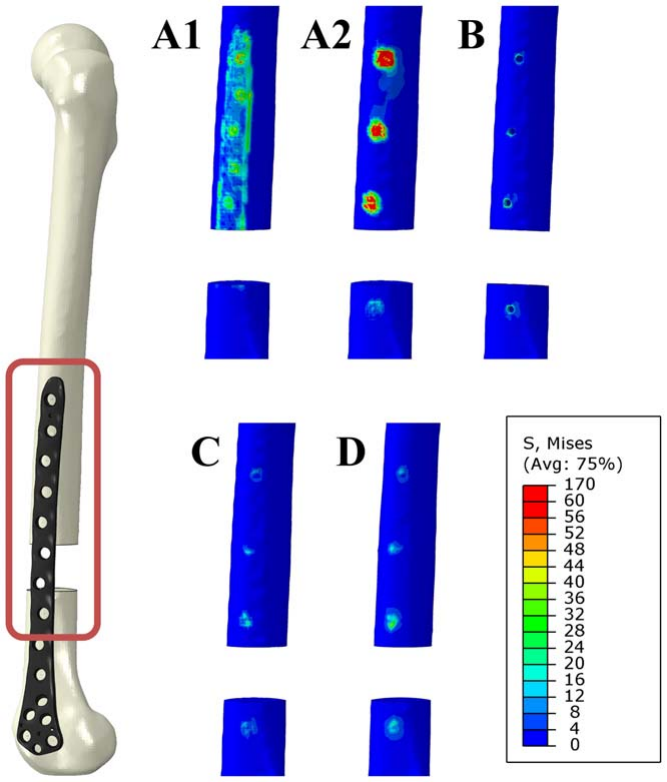
Stress distribution within the area between the bone and the osteosynthesis plate. Results are shown on the surface of the bone (marked with red rectangle on the right). An upper limit of 60 MPa was set for comparison of all cases. Stresses are shown on the lateral site of the femur without the osteosynthesis plate for both tied contact models (Case A1 and A2), the model concerning the screw holes (Case B) and both models with the implemented structural screws (Case C and D).

Subsequently, the stress analysis compared the von Mises equivalent stresses. Highest stresses occurred at the proximal end of the gap for all numerical models and are shown as stress distribution in Figure 12. For all screw models stresses occurred only within the surrounding area of the screws. In contrast to that, stresses occurred within the whole contact area between the osteosynthesis plate and the femoral bone for both tied fixations, influencing a larger area for the two than for the seven contact fixation method. Both direct fixation models showed the highest amount of stress in the area around the screw holes of the osteosynthesis plate.

## Discussion

In this study we described the accuracy of different numerical techniques to consider the fixation of an osteosynthesis implant to the bone stock in comparison to experimental testing. Good correlations in both femoral head displacement and gap alteration were found between experimental testing with an accuracy of approximately 95% for the FE models concerning screws. However, results obtained for the gap alteration of 1.78 mm under the applied load of 227 N did not represent a micromotion, as the investigated model is a large segmental defect model and therefore, the results are more an indicator for the stability of the osteosynthesis system.

The test setup for the biomechanical application and screw implementation was performed similar to testing procedures reported in studies with focus on the comparison of different types of internal plate fixation [3]–[5]. Validation was conducted using a composite femur due to the consistent and reproducible material properties compared to human specimens [24], [25]. Furthermore, the overall behaviour of the composite femur is similar to that of human bone and is therefore an established method for biomechanical testing [26], [27]. Nevertheless, specific results, e.g. pull-out testing of screws may differ from human bone.

In addition to the validation of our numerical model we could also show the strong influence of the numerical implant fixation technique on the behaviour under static loading. Especially for clinical predictions and the application of osteosynthesis plates to fractured bones or bones with segmental defects the use of an adequate fixation technique is important for the quality of the numerical results.

The modelling technique of implant fixation for both tied contact models leads generally to too small deformations due to a stiffening of the bone-plate-interface, whereby the seven-contact-model was less stiff and thereby closer to the reality than the two-contact-model. In general, these results suggest that the use of tie fixation techniques leads to inadequate displacements when used with large contact areas and might be an acceptable method for small contact areas with only small deformations. Taylor, et al. [9] used this kind of fixation to fix a Thrust Plate Prosthesis to the bone without modelling additional screws. The tying of bone and implant surfaces will result in increased and non-physiological stresses within the contact surface. Especially for the described bone remodelling processes the impact of this effect remains uncertain. For this reason, modelling of screws in conjunction with the use of osteosynthesis systems and big contact areas is highly recommended.

Concerning the presence of screws our FE simulations showed a good agreement between numerical results and the experimental testing. The use of solid elements is the most common method to consider the screws [6]–[8], [15]. Cegonino, et al. [6] determined the mechanical stability of fractures at the distal femur and different types of implants in a clinical situation similar to our case. In contrast to other authors, hexahedral elements instead of tetrahedral elements were used to discretise the FE models in our study. Nevertheless, for different kinds of implants or screw orientations meshing of the femur and the screws has to be performed individually for each case, which leads to high pre-processing costs.

While the fixation of FE models in the field of non-union in long bones is performed mainly with volumetric elements the mixture of volumetric and structural elements used for the fixation is only reported in a few studies. Seral, et al. [10] used bar elements for the fixation of an intramedulluary osteosynthesis as a support at the distal end to the main fixation in the proximal area with volumetric elements.

Complete fixation of an implant to an open wedge tibial osteotomy was performed by Blecha, et al. [18] using structural beam elements. Although their investigations imply good results, experimental testing for validation purposes is seldom performed. In contrast, Wilke, et al. [17] performed a validation and showed a good correlation between numerical analysis and in-vitro testing with a fixation composed of only structural beam elements for cervical plates.

In the presented studies structural elements were used for the fixation of small implants only [17], [18] or in addition to solid screws as supporting elements for partial fixation [10], whereas the influence of the screws were not clearly determined. In our present study we could show the accuracy of solely used structural elements for the fixation of big-sized implants and large screw-bone-connectivity. Thereby, results of both models, Case C & D with the structural screws and Case B using solid screws, showed differences of approximately 1%. Therefore, the use of structural elements could replace solid screws and thereby spare the modelling effort for the complex screw structures and the consideration of screw holes, which complicates the meshing of the bone, especially when the mesh is composed of hexahedral elements.

Structural elements like beams are a common method to discretise slender structures, e.g. in external fixation systems with satisfying accuracy [28], [29]. The described method can therefore be used to create screws parameterised only by few parameters, e.g. radius of influence, length and direction on FE meshes with minimal effort. Due to the use of structural beam and rigid connector elements the screws could be implemented automatically in arbitrary FE models after volume mesh generation. For this study complete screw modelling could be performed in less than one minute. Therefore, numerous variations of screw parameters, e.g. screw radius, length, orientation, amount of screws in combination with different osteosynthesis systems can be investigated in order to give clinically relevant propositions.

Besides the different types of screw modelling, we could also show the influence of the element type used for the meshing of the femur (hexahedral versus tetrahedral). Both meshes consisted of a global edge length of approximately 2 mm as recommended to be in good convergence [21], [22]. Our own testing showed a convergence of the gap alteration at this edge length. Furthermore, using second-order ten-node tetrahedral elements should be used for accurate stress distribution within the elements [22]. It should be noted, both meshes performed in a similar way but took different computational time due to the different DOF. Therefore, hexahedral elements showed an optimum of result accuracy and computational time. Hence this numerical model using hexahedral elements is suitable to be used for further numerical investigations and the developed method can be used for the screw generation in arbitrary FE models.

As the results showed good agreement between experimental testing and numerical analysis this kind of screw modelling approach and the CT-based material distribution can be also used for patient-specific human bones with sufficient accuracy.

In addition, more complex physiological boundary conditions can be investigated, e.g. considering muscle forces and loads from daily routines like walking, stair climbing or stumbling in order to determine the mechanical behaviour under every-day situations.
